# Prognostic performance of the Simplified Acute Physiology Score II in major Croatian hospitals: a prospective multicenter study

**DOI:** 10.3325/cmj.2012.53.442

**Published:** 2012-10

**Authors:** Kristian Deša, Mladen Perić, Ino Husedžinović, Alan Šustić, Anđelko Korušić, Vjekoslav Karadža, Dražen Matleković, Branka Prstec-Veronek, Marta Žuvić-Butorac, Jadranko Sokolić, Mladen Širanović, Danica Bošnjak, Jasna Špiček-Macan, Denis Guštin, Draženka Ožeg-Jakopović

**Affiliations:** 1 Department of Anesthesiology and Intensive Care Medicine, Rijeka University Hospital, Rijeka, Croatia; 2 Department of Anesthesiology and Intensive Care Medicine, “Sestre milosrdnice” University Hospital, Zagreb, Croatia; 3 Department of Anesthesiology and Intensive Care Medicine, Dubrava University Hospital, Zagreb, Croatia; 4 Department of Anesthesiology and Intensive Care Medicine, Jordanovac University Hospital for Lung Diseases, Zagreb, Croatia; 5 Department of Anesthesiology and Intensive Care Medicine, Merkur University Hospital, Zagreb, Croatia; 6 Department of Anesthesiology and Intensive Care Medicine, Varaždin General Hospital, Varaždin, Croatia; 7 Faculty of Engineering, University of Rijeka, Rijeka, Croatia

## Abstract

**Aim:**

To perform an external validation of the original Simplified Acute Physiology Score II (SAPS II) system and to assess its performance in a selected group of patients in major Croatian hospitals.

**Methods:**

A prospective, multicenter study was conducted in five university hospitals and one general hospital during a six-month period between November 1, 2007 and May 1, 2008. Standardized hospital mortality ratio (SMR) was calculated from the mean predicted mortality of all the 2756 patients and the actual mortality for the same group of patients. The validation of SAPS II was made using the area under receiver operating characteristic curve (AUC), 2 × 2 classification tables, and Hosmer-Lemeshow tests.

**Results:**

The predicted mortality was as low as 14.6% due to a small proportion of medical patients and the SMR being 0.89 (95% confidence interval [CI], 0.78-0.98). The SAPS II system demonstrated a good discriminatory power as measured by the AUC (0.85; standard error [SE] = 0.012; 95% CI = 0.840-0.866; *P* < 0.001). This system significantly overestimated the actual mortality (Hosmer-Lemeshow goodness-of-fit H statistic: χ^2^ = 584.4; *P* < 0.001 and C statistics: χ^2^_8_ = 313.0; *P* < 0.001) in the group of patients included in the study.

**Conclusion:**

The SAPS II had a good discrimination, but it significantly overestimated the observed mortality in comparison with the predicted mortality in this group of patients in Croatia. Therefore, caution is required when an evaluation is performed at the individual level.

In the recent decades, scoring systems for assessing the severity of disease on admission to intensive care units (ICU) have been used for performance evaluation in different ICUs in different countries. The comparison is based on the calculation of the standardized mortality ratio (SMR) from the mean value of all predicted mortalities and the observed mortality in the same group of patients (1). The SMR calculation method is widely used for a comparison of ICUs that are specialized for the treatment of very different patients with regard to their age, comorbidities, and current condition (the reason for admission and disorder of physiological variables) (2).

One of the most frequently used disease-severity scoring systems, created by Le Gall et al (3) in 1993, is the second version of the Simplified Acute Physiology Score (SAPS II). The SAPS II was developed on the basis of a large number of patients as an upgrade of the first version created by the same authors in 1984 (3,4). As opposed to the SAPS I, the SAPS II resulted from the selection and weighing of each variable by logistic regression. The SAPS II total score is the sum of scores of the worst value for each variable within the first 24 hours after ICU admission. The score is then converted into the probability of dying, that is, predicted mortality, by using the model equation.

The SAPS II has been validated in different populations of ICU patients in different countries. Research on the topic was first performed in the Western countries, and the reported SMRs ranged between 0.7 and 1.2 (5-17). Subsequent studies were mostly performed in single institutions in non-Western countries and reported poorer SMR results (somewhere, SMR were >1.5) (18-22). To the best of our knowledge, only two multicenter studies from non-Western countries have been published, and none from the countries in Eastern Europe (23,24).

The primary aim of the study was to perform an external validation of the SAPS II system in a group of the ICU patients treated in the major hospitals in Croatia. In addition, this study aimed to determine the actual severity of disease on ICU admission, before the Diagnosis Related Groups system is introduced in Croatia, which will require SAPS II score assessment on ICU admission.

## Patients and methods

### Patients

The project “Performance of Intensive Care Medicine in the Republic of Croatia” began in 2007 under the auspices of the Croatian Association for Anesthesiology and Intensive Care Medicine. The prospective study was carried out in five university hospitals and one general hospital between November 1, 2007 and May 1, 2008. The participant hospitals were “Rijeka University Hospital” from Rijeka, “Sestre Milosrdnice,” “Dubrava,” “Jordanovac,” and “Merkur” university hospitals from Zagreb, and “Varaždin General Hospital” from Varaždin.

Before the study, a dedicated computer program was created by the Microsoft Access 2003 (Microsoft, Redmond, WA, USA). Detailed instructions about the program, as well as about the system for mortality prediction, were prepared. Two data entry trainings were held to educate the personnel responsible for entering the data into the database (anesthesiology residents and specialists). The computer program automatically scored each variable, generated alerts about illogical and/or extreme variable values, and excluded patients from the study according to the criteria used in the original study, that is, patients younger than 18 years, patients with burns, patients with coronary disease, heart surgical patients, and patients who were in the ICU for less than 4 hours (3). Furthermore, in the final computer report, estimated mortality was taken into consideration only for the first ICU stay in case the patient was admitted to the ICU more than once during a single hospitalization. Data collection was completed on September 1, 2008 and was all-inclusive.

All variables for the SAPS II scoring system were manually collected, including age, chronic diseases (hematologic malignancies, metastatic cancer, and/or acquired immunodeficiency syndrome), type of admission (elective surgery, emergency surgery, or medical), and physiological variables (body temperature, heart rate, systolic blood pressure, the ratio of partial oxygen pressure and inspired oxygen concentration, diuresis, urea, potassium, sodium, bicarbonates, leukocytes, bilirubin, and Glasgow Coma Score). Medical patients were defined as patients without any surgical procedure within seven days of ICU admission. The consciousness of the patient was evaluated by the Glasgow Coma Scale (GSC), and for the patients who were sedated at the moment of ICU admission the value of GCS had been recorded before sedation started. Among all recorded values for a particular variable within the first 24 hours of ICU stay, the value that had the highest number of points was selected from the patient’s record. If the value was entered as a range, the computer program converted the range to a number of points (3). Except for the variables included in the SAPS II, we measured the length of ICU stay as well as the length of hospital stay. The outcome of interest (alive or dead) was measured at the point of discharge from the hospital. The treatment outcome on discharge from the ICU was measured in the same manner. Since all the patient variables used in this study were regularly collected in everyday work, no additional interventions were needed. The study was approved by the ethics committee of the Rijeka University Hospital Center.

### Statistical analysis

The SAPS II score was calculated for all patients by adding up the number of points for each variable and the probability of death was computed according to the original SAPS II equation (3). The observed mortality was divided by the mean value of all predicted mortalities to calculate the SMR. The 95% confidence intervals (CI) for SMRs were calculated by regarding the observed mortality as a Poisson variable, and then dividing its 95% CI by the predicted mortality (25). The survivors and deceased patients were compared using univariate comparisons. Continuous variables were presented as either means with standard deviation (for normally distributed data) or medians with an interquartile range. Comparisons were performed using either the *t* test or the Mann-Whitney U-test, whichever was suitable. Categorical variables were presented by frequencies and percentages and compared using the χ^2^ test. All statistical tests were two-tailed. *P* < 0.05 was considered statistically significant.

The SAPS II score was validated in a group of patients receiving intensive care medicine in Croatia while testing for discrimination (ability to discriminate between patients who will live and patients who will die) and calibration (degree of agreement between predicted and observed mortality). Discrimination was evaluated by calculating the area under the receiver operating characteristic curve (ROC), with a standard error (SE), 95% CI, and Z statistics. The constructed ROC curve specified a range of probabilities of death, and a 2 × 2 classification table of predicted and observed mortalities was created for each decision criterion, that is, the ROC curve showed the graphic relationship between the sensitivity and specificity. The higher the true-positive frequency in comparison with the false-positive frequency, the larger the area under the ROC curve. The 2 × 2 classification table was created for the three decision criteria of the predicted mortality of 0.1 (10%), 0.5 (50%), and 0.9 (90%), which were compared using the McNamara χ^2^ test (26). Calibration was evaluated by using Hosmer-Lemeshow C and H goodness-of-fit statistics and calibration curve. The patients were divided in 10 groups according to the level of predicted mortality in order to calculate the H value. To calculate the C value, the patients were divided in 10 groups of an equal size, and the predicted mortality was compared with the observed mortality in each of the groups. High C values and low P values (*P* < 0.05) suggested that the model did not predict well the observed mortality (27). When we investigated the uniformity of fit, we used two strategies that compared SMRs and 95% CIs: participant ICUs and the type of the patients. Data were analyzed using MedCalc ver. 11.6.1.0, MedCalc Software (bvba, Mariakerke, Belgium), Statistica ver. 9.1 (StatSoft Inc., Tulsa, OK, USA), and SPSS, version 14.0 (SPSS Inc., Chicago, IL, USA).

## Results

All ICUs included in the study were combined medical/surgical ICUs headed by anesthesiologists, with other specialists on the team. In the participating hospitals, there were 68 ICU beds (range, 7-18) and 3613 acute beds (range, 237-1050). In addition to the ICUs included in this study, there were also other ICUs that were not included in the study, with a total of 102 intensive care beds. Of the total number of acute beds, 4.7% were intensive care beds, that is, the ratio of intensive care to acute beds was 1 to 21. The ratio of mean anesthesiology specialists per ICU during the day was 1.8 (range 1-3) and during the night 1.2 (range 1-2). The nurse to bed ratio was 0.5 (range 0.4-0.7) during the day as well as during the night.

We analyzed the data on 3572 patients who were admitted to ICUs during the study period. Exclusion criteria were met by 814 patients, who were mostly heart surgical patients (n = 435). In addition, two patients were excluded from the final analysis due to incomplete data. The final analysis included 2756 patients. The average number of patients per ICU was 459 (range 314-596)

The median age of the patients was 64 (range 52-73) years, and 61% were men. According to the type of admission, most patients were admitted after elective surgery, followed by patients admitted after an emergency surgery, and medical patients (62%, 29%, and 9%, respectively). The median length of stay in the ICU was 2 (range 2-4) and the median length of hospital stay was 11 (8-17) days (Table 1).

**Table 1:** Basic descriptive characteristic of patients and a comparison between survivors and non-survivors in intensive care units (ICU) in major Croatian hospitals*

Characteristic	All	Survivors	Non-survivors	*P*
Number of patients	2756	2399	357	
Age (y)	64 (52-73)	62 (50-72)	71 (59-78)	<0.001
Sex (n, %):				
male	1680 (61)	1479 (88)	201 (12)	0.408
female	1076 (39)	920 (86)	156 (15)	
Type of admission (n, %):				
medical	245 (9)	153 (62)	92 (38)	<0.001
emergency surgery	808 (29)	624 (77)	184 (23)	
elective surgery	1703 (62)	1622 (95)	81 (5)	
Length of stay (days):				
ICU	2 (2-4)	2 (2-4)	4 (2-8)	<0.001
hospital	11 (8-17)	11 (9-16)	9 (4-19)	0.244
Simplified Acute Physiology Score II	24 (16-37)	21 (15-30)	50 (34-73)	<0.001

*Data are presented either as medians with a q25-q75 range or as a number of patients (n, %).

The survivors were younger than the patients who died, whereas sex had no effect on survival (Table 1). The type of admission had a significant influence on the outcome of hospital treatment, with medical patients having a higher mortality than surgical ones. Emergency surgical patients had a higher mortality than elective surgical patients. The survivors had a shorter ICU stay than the patients who died, whereas the length of hospital stay of survivors and deceased patients did not differ (Table 1).

On ICU admission, the median of SAPS II score was 24 (range, 16-37) and the predicted mortality was 14.6%. Most patients had a low predicted mortality, that is, the predicted mortality was lower than 10% in 67% of the patients; 61.3% patients had an SAPS II score less than 27. The predicted mortality varied between the hospitals (range 5.8%-27.4%), and survivors had a lower predicted mortality on admission than patients who died (10.1% vs 44.9%, respectively; *P* < 0.001; Table 1).

The observed ICU mortality was 8.1% and the hospital mortality of patients included in the study was 13%. Similar to the predicted mortality, the observed mortality also varied across hospitals, from 2.1% to 23.8%. The total SMR was 0.89 (95% CI 0.78-0.98). The SMR in one of the hospitals was 0.36 (95% CI 0.16-0.70), deviating from the SMRs in other hospitals. There were no big deviations in SMRs among other hospitals (1.08, 0.95, 0.86, 0.84, 0.87, and 0.89), and 95% CI in all other hospitals included number 1 (0.86-1.33, 0.70-1.24, 0.64-1.12, 0.61-1.12, 0.71-1.04, and 0.79-0.98). Regarding the type of patients, the lowest SMR was for elective surgery patients (0.82, 95% CI 0.62-0.97), followed by emergency surgery (0.91, 95% CI 0.77-1.18) and medical patients (0.98, 95% CI 0.78-1.18).

**Figure 1:**
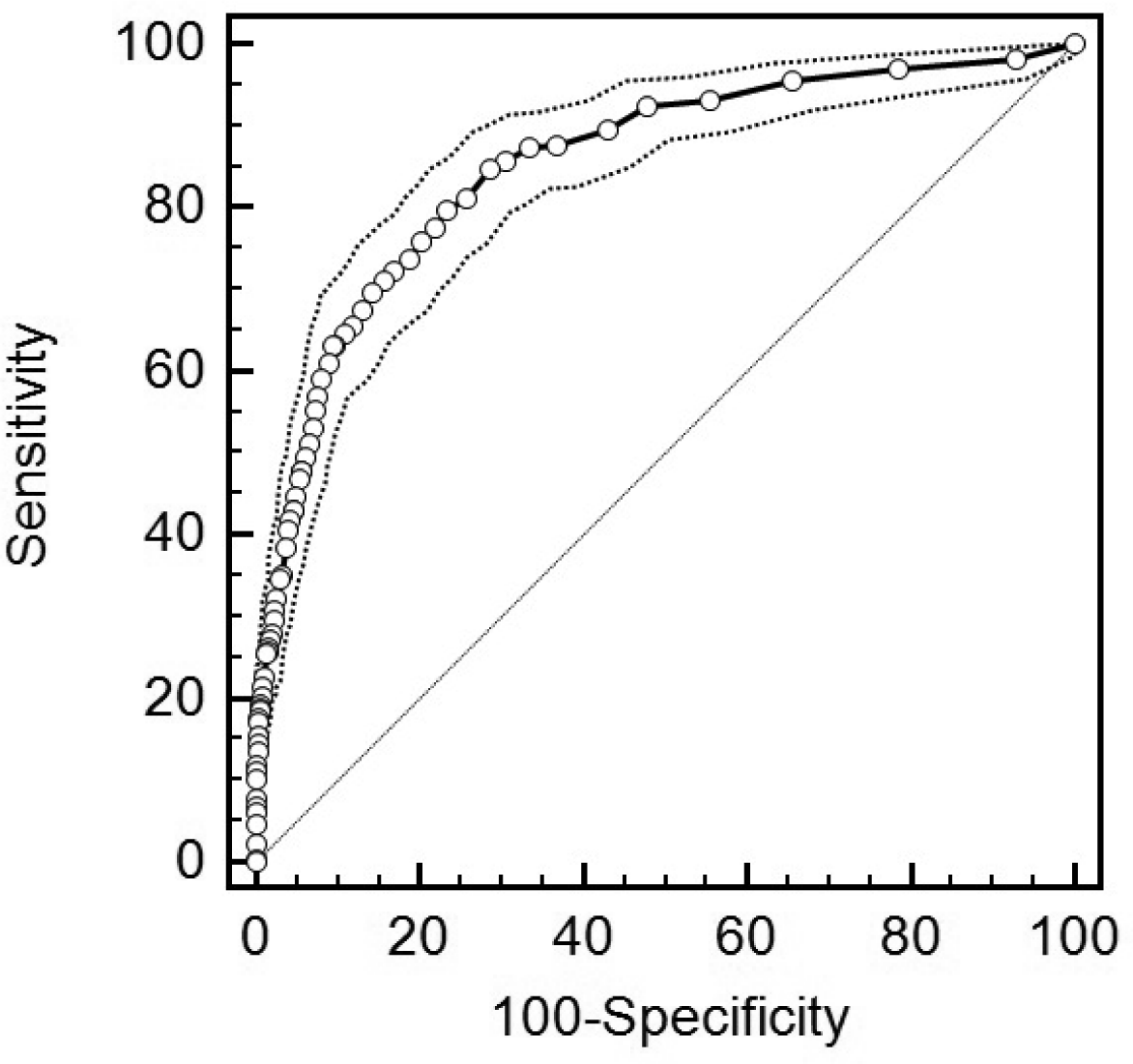
Receiver operating characteristic (ROC) curve for the Simplified Acute Physiology Score II (curved line connected with the circles) including standard deviation lines (curved dotted lines) in intensive care units (ICU) in major Croatian hospitals and example of ROC value of 0.5 (diagonal line). The relationship between true positives (sensitivity) and false positives (100-specificity) is presented. The area under the ROC curve was 0.85.

The discriminatory power of the SAPS II system, as assessed by the area under the ROC curve, was 0.85 (SE = 0.012; 95% CI = 0.840–0.866; *P* < 0.001; Figure 1). With the decision criteria of 10%, 50%, and 90%, the sensitivity was 81.2%, 43.0%, and 11.8% and the false-positive rate was 25.9%, 4.6%, and 4.6%, respectively (Table 2).

**Table 2:** Classification table for Simplified Acute Physiology Score II, with decision criteria of 0.1, 0.5, and 0.9 of predicted mortality (CI – confidence interval)*

Decision criterion	Predicted to live	Predicted to die
10%:		
observed survivors (n)	231	2168
observed non-survivors (n)	46	311
sensitivity (95% CI)	81.2 (76.8-85.1)	
specificity (95% CI)	74.1 (72.3-75.9)	
positive likelihood ratio (95% CI)	3.1 (3.0-3.3)	
negative likelihood ratio (95% CI)	0.25 (0.2-0.3)	
overall correct classification (95% CI)	0.20 (0.19-0.21)	
50%:		
observed survivors (n)	1191	1208
observed non-survivors (n)	160	197
sensitivity (95% CI)	43.1 (37.9-48.5)	
specificity (95% CI)	95.4 (94.5-96.2)	
positive likelihood ratio (95% CI)	9.3 (8.3-10.5)	
negative likelihood ratio (95% CI)	0.60 (0.5-0.7)	
overall correct classification (95% CI)	0.50 (0.48-0.51)	
90%:		
observed survivors (n)	2311	88
observed non-survivors (n)	220	137
sensitivity (95% CI)	11.8 (8.6-15.6)	
specificity (95% CI)	99.8 (99.5-99.9)	
positive likelihood ratio (95% CI)	70.6 (53.1-93.7)	
negative likelihood ratio (95% CI)	0.88 (0.3-2.4)	
overall correct classification (95% CI)	0.89 (0.88-0.91)	

*McNemar χ^2^ test for 0.1, 0.5, and 0.9 decision criteria, *P* < 0.001.

**Figure 2:**
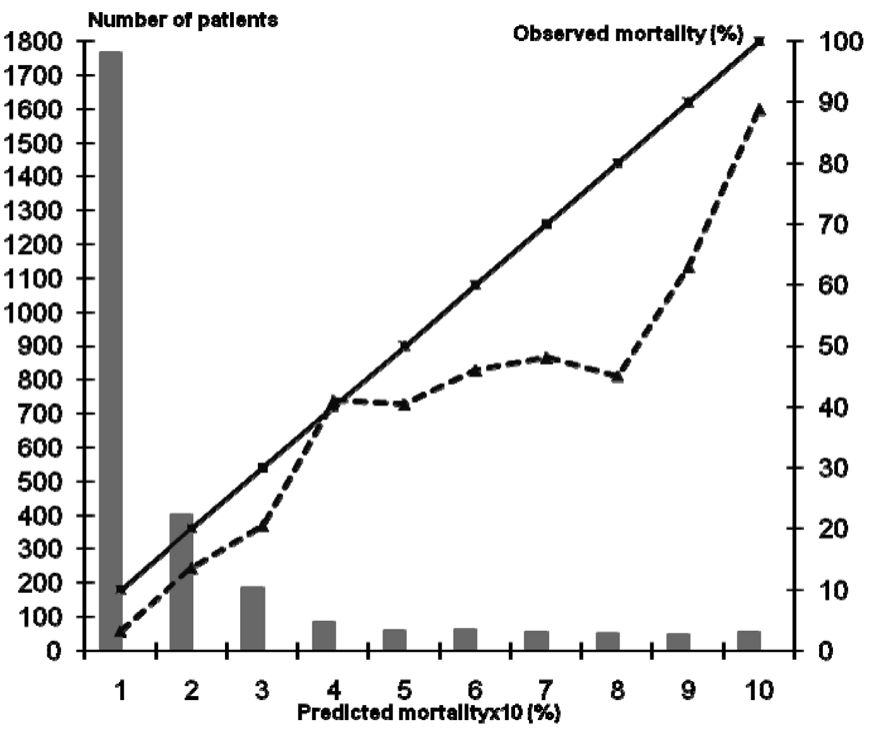
Calibration curve for the Simplified Acute Physiology Score II. The solid line represents a perfect correspondence between the observed and predicted mortality; the dotted line represents the observed vs predicted mortality; columns show the distribution of the patients in the analyzed groups.

The calibration was tested using the Hosmer-Lemeshow goodness of fit H test (χ^2^_8_ = 584.4; *P* < 0.001) and C test (χ^2^_8_ = 313.0; *P* < 0.001). The difference between the predicted and observed mortality was significant (Tables 3 and 4). The calibration curve of the SAPS II system in this group of ICU patients treated in major Croatian hospitals indicated the lower observed mortality in comparison with the predicted mortality in all groups except for the group whose predicted mortality was 40%. The deviation from the ideal (full line) is larger in groups of patients with a higher predicted mortality (Figure 2).

**Table 3:** Hosmer-Lemeshow goodness-of-fit test H for Simplified Acute Physiology Score II. The table is collapsed on fixed values of the estimated probabilities (statistics: χ^2^ = 584.4, *P* < 0.001)

Predicted probability	Observed and expected frequencies by deciles (n)				Total (n)
	observed survivors	expected survivors	observed deaths	expected deaths	
0.00-0.10	231	265.771	46	11.229	277
>0.10-0.20	234	254.501	36	15.499	270
>0.20-0.30	240	253.425	34	20.575	274
>0.30-0.40	189	199.198	31	20.802	220
>0.40-0.50	297	268.910	13	41.090	310
>0.50-0.60	272	235.181	27	63.819	299
>0.60-0.70	184	138.415	15	60.585	199
>0.70-0.80	347	233.816	5	118.184	352
>0.80-0.90	317	210.610	13	119.390	330
>0.90-1.00	88	59.722	137	165.278	225
	2399		357		2756

**Table 4:** Hosmer-Lemeshow goodness-of-fit test C for Simplified Acute Physiology Score II. The table is collapsed on fixed values of the estimated probabilities (statistics: χ^2^ = 313.0, *P* < 0.001)

Predicted probability	Observed and expected frequencies by deciles (n)				Total (n)
	observed survivors	expected survivors	observed deaths	expected deaths	
0.00-0.010	260	288.987	42	13.013	302
>0.010-0.014	184	197.803	25	11.197	209
>0.010-0.022	261	257.655	13	16.345	274
>0.022-0.035	297	297.442	25	24.558	322
>0.035-0.050	150	164.550	31	16.450	181
>0.050-0.080	255	235.329	14	33.671	269
>0.080-0.120	347	308.154	29	67.846	376
>0.120-0.200	364	282.465	18	99.535	382
>0.200-0.390	224	170.329	50	103.671	274
>0.390-1.000	57	27.642	110	139.358	167
	2399		357		2756

## Discussion

We reported the results of a prospective, multicenter study performed in five university hospitals and one general hospital in Croatia. Multi-purpose scoring systems for predicting mortality based on the SMR are the only methods that allow for a comparison of intensive care treatment results in different patient groups in different countries and ICUs. So far, many studies have been published in countries with different social, demographic, economic, and medical conditions (5-24). In our study, SAPS II system showed good discrimination and poor calibration properties.

Patients treated in major Croatian hospitals were not significantly different according to their demographic characteristics from those included in the studies performed in Western countries (5-17). The median age of patients included in the study was 64 years, and two-thirds were men. The reason for a lower percentage of medical patients, which differed from that in the original study and other studies (5-17), was that the ICUs included in the study were managed by anesthesiologists who primarily dealt with surgical patients. In all the studied hospitals, the ICUs that were not included in the study were managed by physicians of other specialties, such as cardiologists or neurologists, who treated mostly medical patients.

In comparison with the studies performed in Western countries, the group of the patients included in our study had a low SAPS II score and a low predicted mortality. The low proportion of medical patients was the reason why we recorded low predicted mortality on admission. According to the type of admission, most patients included in the study were elective surgical patients. These patients are usually admitted to the ICU for a 24-hour supervision and are characterized by a low predicted mortality. Accordingly, the lengths of ICU and hospital stays were shorter than those reported in the literature (5-17). In addition, the observed ICU mortality and in-hospital mortality were low in comparison with those reported in Western countries (5-17).

The influence of different variables on survival was similar to that reported in Western countries (5-17). The survivors were younger and had a lower predicted mortality, and there was no difference in survival between men and women. On the other hand, the highest mortality was recorded in medical patients, followed by emergency surgical patients and elective surgical patients. The survivors had a shorter length of ICU stay, while the length of hospital stay was similar in survivors and deceased patients.

The SMR was 0.89 (95% CI 0.79-0.98) and deviated toward a lower value in only one hospital: a specialized hospital for thoracic surgical patients where all operated patients are admitted to the ICU by default. Since SMR and 95% CI for elective surgical patients deviated from emergency surgical and medical patients, results from this hospital were probably the reason for the overall lack of uniformity of fit, because elective surgical patients were mostly treated in this hospital (93%). Other hospitals did not differ in SMRs and 95% CIs, which indicated a similar uniformity of fit in these hospitals. In addition, the total SMR was within the range reported in Western countries (5-17). However, the results of these studies should be interpreted with caution, because they were reported approximately 10 years ago, and changes in population and treatment over time may change the prognosis of patients, thereby limiting the applicability of prognostic models (28).

The validation of the SAPS II system in this group of ICU patients in Croatia showed good discriminative properties. The SAPS II system discriminated well between the patients who would live and those who would die, which is similar to the data from Western countries. The 2 × 2 classification table showed low sensitivity values, low-false positive rates, and low overall correct classification for all decision criteria. Such a finding was already reported in the literature, where the analyzed sample of patients included a large proportion of those with a low predicted mortality (29). Our calibration tests confirmed in a Croatian ICU population that the SAPS II system predicted a mortality that was higher than the observed one. This calls for caution when assessment is made at an individual level. Improved prediction at an individual level may be achieved by the customization of the SAPS II system for patients treated in Croatian hospitals, as shown in previous studies (6,12,16). The fact that ICUs included in the study were managed by anesthesiologists, who primarily dealt with surgical patients, could be considered a limitation. However, SAPS II was created to overcome the differences between ICU patients by focusing on the severity rather than the type of the disease.

SAPS II could be a very useful tool for benchmarking, which includes a comparison between similar ICUs. Benchmarking has been recently included among indicators for improving the safety and quality of care for intensive care patients (30). In this sense, it is necessary to create a national database of intensive care outcomes, compare the existing ICUs, find ICUs of excellent practice, and spread it all over the country (31).

The SAPS II system can discriminate well between the patients who will survive and those who will die. However, this system overestimates the mortality of the analyzed group of ICU patients; therefore, the prognosis in individual patients has to be made with caution.

## References

[1] Ridley S (1998). Severity of scoring systems and performance appraisal.. Anaesthesia.

[2] Knaus WA, Draper EA, Wagner DP, Zimmerman JE (1986). An evaluation of outcome from intensive care in major medical centers.. Ann Intern Med.

[3] Le Gall JR, Lemeshow S, Saulnier F (1993). A new simplified acute physiology score (SAPS II) based on European/North American multicentre study.. JAMA.

[4] Le Gall JR, Loirat P, Alperovitch A, Glaser P, Granthil C, Mathieu D (1984). A simplified acute physiology score for ICU patients.. Crit Care Med.

[5] Castella X, Artigas A, Bion J, Kari A (1995). A comparison of severity of illness scoring systems for intensive care unit patients: Results of a multicenter, multinational study.. Crit Care Med.

[6] Apolone G, Bertolini G, D'Amico R, Iapichino G, Cattaneo A, De Salvo G (1996). The performance of SAPS II in cohort of patients admitted to 99 Italian ICUs: Results from GiViTi.. Intensive Care Med.

[7] Moreno R, Apolone G, Miranda DR (1998). Evaluation of the uniformity of fit of general outcome prediction models.. Intensive Care Med.

[8] Moreno R, Morais P (1997). Outcome prediction in intensive care: results of prospective, multicentre Portuguese study.. Intensive Care Med.

[9] Moreno R, Miranda DR, Fidler V (1998). Schilfgaarde. Evaluation of two outcome prediction models on an independent database.. Crit Care Med.

[10] Bertolini G, Amico RD, Apolone G, Cattaneo A, Ravizza A, Iapichino G (1998). Predicting outcome in the intensive care unit using scoring systems – is new better? A comparison of SAPS and SAPS II in a cohort of 1393 patients.. Med Care.

[11] Tan IK (1998). APACHE II and SAPS II are poorly calibrated in a Hong Kong intensive care unit.. Ann Acad Med Singapore.

[12] Metnitz PGH, Valentin A, Vesely H, Lang T, Lenz K, Steltzer H (1999). Prognostic performance and customization of the SAPS II: results of a multicenter Austrian study.. Intensive Care Med.

[13] Livingston BM, MacKirdy FN (2000). Assessment of the performance of five intensive care scoring models within a large Scottish database.. Crit Care Med.

[14] Markgraf R, Deutschinoff G, Pientka L, Scholten T (2000). Comparison of acute physiology and chronic health evaluations II and III and simplified acute physiology score II: a prospective cohort study evaluating these methods to predict outcome in a German interdisplinary intensive care unit.. Crit Care Med.

[15] Katsaragakis S, Papadimitropoulos K, Antonakis P, Strergiopoulos S, Konstadoulakis M, Androulakis G (2000). Comparison of Acute Physiology and Chronic Health Evaluation II (APACHE II) and Simplified Acute Physiology Score II (SAPS II) scoring systems in a single Greek intensive care unit.. Crit Care Med.

[16] Harrison DA, Brady AR, Parry GJ, Carpenter JR, Rowan K (2006). Recalibration of risk prediction model in a large multicenter cohort of admissions to adult, general critical care units in the United Kingdom.. Crit Care Med.

[17] Villers D, Fulgenicio JP, Gouzes C, Hemery F, Bleriot JP, Garrigues B (2006). ICU performance: results of French Study involving 80.000 ICU stays.. Ann Fr Anesth Reanim.

[18] Vosylius S, Sipylaite J, Ivaskevicius J (2004). Evaluation of intensive care unit performance in Lithuania using SAPS II system.. Eur J Anaesthesiol.

[19] Kim EK, Kwon YD, Hwang JH (2005). Comparing the performance of three severity scoring systems for ICU patients: APACHE III, SAPS II, MPM II.. J Prev Med Pub Public Health.

[20] Ratanarat R, Thanakittiwirun M, Vilaichone W, Thongyoo S, Permpikul C (2005). Prediction of mortality by using standard scoring systems in a medical intensive care unit in Thailand.. J Med Assoc Thai.

[21] Aggarwal AN, Sarkar P, Gupta D, Jindal SK (2006). Performance of standard severity scoring systems for outcome prediction in patients admitted to a respiratory intensive care unit in North India.. Respirology.

[22] Khwannimit B, Geater A (2007). A comparison of APACHE II and SAPS II scoring systems in predicting hospital mortality in Thai adult intensive care units.. J Med Assoc Thai.

[23] Nouira S, Belghith M, Elatrous S, Jaafoura M, Ellouzi M, Boujdaria R (1998). Predictive value of severity scoring systems: Comparison of four models in Tunisian adult intensive care units.. Crit Care Med.

[24] Hariharan S, Chen D, Merrit-Charles L, Bobb N, De Freitas L, Esdelle-Thomas A (2007). An evaluation of the intensive care unit resources and utilization in Trinidad.. West Indian Med J.

[25] Goldhill DR, Sumner A (1998). Outcome of intensive care patients in a group of British intensive care patients.. Crit Care Med.

[26] Hanley JA, McNeil BJ (1982). The meaning and use of the area under receiver operating characteristic (ROC) curve.. Radiology.

[27] Lemeshow S, Hosmer DW (1982). A review of goodness of fit statistics for use in the development of logistic regression models.. Am J Epidemiol.

[28] Minne L, Eslami S, de Keizer N, de Jonge E, de Rooij SE, Abu-Hanna A (2012). Effect of changes over time in the performance of customized SAPS II model on the quality care assessment.. Intensive Care Med.

[29] Lemeshow S, Le Gall J-R (1994). Modeling the severity of illness of ICU patients. A systems update.. JAMA.

[30] Rhodes A, Moreno RP, Azoulay E, Capuzzo M, Chiche JD, Eddelston J (2012). Prospectively defined indicators to improve the safety and quality of care for critically ill patients: a report from the Task Force on Safety and Quality of the European Society of Intensive Care Medicine (ESCIM).. Intensive Care Med.

[31] Glance LG, Szalados JE (2002). Benchmarking in critical care: the road ahead.. Chest.

